# Transvaginal Laparoscopic Appendectomy: A Systematic Review

**DOI:** 10.7759/cureus.51962

**Published:** 2024-01-09

**Authors:** Ethan Slouha, Stefan J Biput, Brandon Krumbach, Lucy A Clunes, Theofanis F Kollias

**Affiliations:** 1 Anatomical Sciences, St. George's University School of Medicine, St. George's, GRD; 2 Medicine, St George's University School of Medicine, St. George's, GRD; 3 Anatomy, St. George’s University School of Medicine, St. George's, GRD; 4 Pharmacology, St. George’s University School of Medicine, St. George's, GRD; 5 Microbiology, Immunology, and Pharmacology, St. George's University School of Medicine, St. George's, GRD

**Keywords:** surgical procedures, appendicitis, notes surgery, laparoscopic appendectomy, transvaginal approach

## Abstract

Appendectomy remains the gold standard for treating appendicitis, but advancements in laparoscopic techniques have shifted the paradigm. Natural orifice transluminal endoscopic surgery (NOTES) and transvaginal appendectomy (TVA) offer a potentially less invasive alternative to traditional laparoscopic appendectomy (LA). This article systematically reviews the procedures, perceptions, and complications of TVA to assess its viability as a surgical option. Between January 1, 2003, and November 1, 2023, 4832 case reports, case series, and experimental and observational peer-reviewed publications were examined and filtered using the keyword "Transvaginal Laparoscopic Appendectomy." The publications were screened using PRISMA guidelines, and 20 studies were included for analysis and review. Survey results showed that women's acceptance of TVA was 43%, citing reduced invasiveness as a major reason for positive reception. TVA procedures exhibited consistency, with variations in appendectomy methods, appendix removal, and posterior fornix incision closure. Positive outcomes included shorter operation times, reduced postoperative pain, and minimal scarring. Complications were uncommon but included bladder puncture, urinary tract infections, and intra-abdominal abscesses. Indications primarily focused on surgical safety, reduced scarring, and postoperative benefits. Sexual function post-TVA exhibited no significant differences in most cases, with a recovery period of two to four weeks. This systematic review suggests that TVA is a promising alternative to traditional LA, offering potential advantages in terms of postoperative complications. While the existing literature indicates positive outcomes, further research with larger sample sizes and long-term follow-ups is needed to validate the efficacy and safety of TVA and assess how the procedure impacts the reproductive function of patients.

## Introduction and background

Appendectomy

Appendectomy is the gold standard of treatment for appendicitis; other forms of treatment in patients that are not critical or are uncomplicated could be managed with antibiotics [1,2]. Following the first 36 hours from the beginning of symptoms, the perforation rate can be anywhere between 16% and 36%, which is accounted for in the surgeon’s decision [3]. When patients are critical, such as a perforated appendix, surgery is recommended and performed relatively close to the diagnosis [1]. Broad-spectrum antibiotics are sometimes used for prophylaxis prior to surgery, but this has been observed to be based on the surgeon’s preference [1]. The anatomical location of the appendix is posterior to the greater omentum and anterior to the iliopsoas muscle and lumbar plexus in the right lower quadrant of the abdomen and supplied by the appendicular artery [2]. Open appendectomy (OA) is the gold standard, where the surgeon performs a Rocky-Davis or Elliot incision close to McBurney’s point and splits the muscles to open the patient up [1-3]. In the last two decades, the rates of OAs have drastically decreased with the intervention of laparoscopic appendectomy (LA) [3].

LA has become the preferred approach as it has been shown to have a lower incidence of wound infection, shorter postoperative hospital stays, and decreased need for analgesic treatment [1-3]. LA requires a pneumoperitoneum to allow for visualization and movement of the equipment within the abdominal cavity [2]. LA is the preferred route in specific subsets of patients such as pediatric patients, pregnant women, obese individuals, and older adults [2]. Some drawbacks to LA are the increased operation time attributed to the setup process and the need for specialized equipment [1,3]. An OA may be preferred if there are complications like an advanced infection or an abscess [1]. A complication like a perforated appendix can still be done laparoscopically [1]. Intra-operative findings such as diffuse peritonitis or appendicular abscess are known predictors of conversion from an LA to an OA [1]. Overall, appendectomies are considered a relatively safe procedure with a low mortality and morbidity rate associated with the event of perforation and stage of the disease [3]. Complications following surgery do occur, however, like wound infection, stump appendicitis, and intra-abdominal abscess [2,3]. As of late, alternative surgical approaches have been created and evaluated, specifically natural orifice transluminal endoscopic surgery (NOTES), to improve appendectomy outcomes further [1].

Natural orifice transluminal endoscopic surgery

NOTES was first mentioned in the 1940s and utilizes flexible endoscopes to enter ulterior routes such as the gastrointestinal or vaginal tract [2,4]. The goal was to create a reproducible and safe technique to gain access to the operating field, including minimal tissue injury, good exposure, safety, and the ability to maintain a seal and manipulate the instrument [2,4,5]. Another aim was to perform surgery without skin incision through endoscopic and laparoscopic techniques [5]. The most preferred route originally was the stomach, transgastric approach, but studies have also focused on transvaginal and transrectal [2,5]. The transgastric approach requires the introduction of the endoscope into the mouth and passing it to the stomach, followed by the puncturing of the stomach wall to access the peritoneal cavity [4,6]. In 2007, the first NOTES procedure performed on humans was done, specifically a transvaginal cholecystectomy [5].

Initial closure of sites like the vagina is done reasonably safely with simple sutures, but ongoing research suggests closure devices using varying mechanical devices or T-tags are promising [5]. Closure must ensure no subsequent leak and anastomotic breakdown [5]. NOTES can only be accomplished with a multichanneled NOTES platform or flexible endoscope that can bend at more than two axes while maintaining stability once in the correct position [5]. The flexibility of the scope allows for remote areas of the peritoneal cavity to be accessed more quickly and easily [6]. A complication with the NOTES is possibly poor spatial orientation, but this can be overcome with an experienced operator [5]. NOTES does run the risk of causing iatrogenic injury, leading to both immediate and delayed complications [4,5]. There is an increased risk of infection, visceral injury, bleeding, and entry site links or delayed anastomosis, which can transform into disastrous results [4,5]. Long-term complications occur in some patients, including adhesion formation and dyspareunia [5].

Aim

With the need to constantly improve how surgeries are performed to improve the patient's outcomes, the appendectomy is no different. NOTES is the new concept that may lead to cosmetic improvement, reduced operation time, decreased pain following surgery, and even reduced hospital stay. One of the novel NOTES approaches is transvaginal, which has led to surgeons experimenting with transvaginal appendectomy (TVA). The goals of the TVA are no different than those of NOTES, and whether it accomplishes this goal has been evaluated through single-case experiments and larger populations. This article aims to review the perceptions, procedures, outcomes, and complications of TVA to emphasize that further research should be done for continuous improvement of current surgical approaches.

## Review

Methods

The current systematic review was performed with stringent adherence to the PRISMA guidelines. Per protocol, a methodical and conclusive inquiry of the existing literature was done using ProQuest, ScienceDirect, and PubMed between January 1, 2003, and November 1, 2023. The keyword used to conduct the inquiry was "Transvaginal Laparoscopic Appendectomy" and was chosen specifically to acquire all case reports, case series, and experimental and observational peer-reviewed publications. The preliminary exploration of the databases used resulted in 4382 publications. Publications produced in another language than English, published before 2003, and duplicates were excluded. After the automatic screening, the newly populated publications were manually evaluated with consideration of their title, study, abstract, and full-text availability. The final step of the screening process involved evaluating the text and correlating it with the chosen keyword, narrowing down the publications to those evolving around the aim of this review. A total of 20 publications were reviewed according to the following criteria:

Inclusion Criteria

The procured publications were elected to full-text analysis based on the following criteria: studies focusing on the application and indication of TVA, case reports, case series, studies performed on humans, publications between 2003 and 2023, full-text availability, and peer-reviewed observational and experimental studies.

Exclusion Criteria

**Figure 1:**
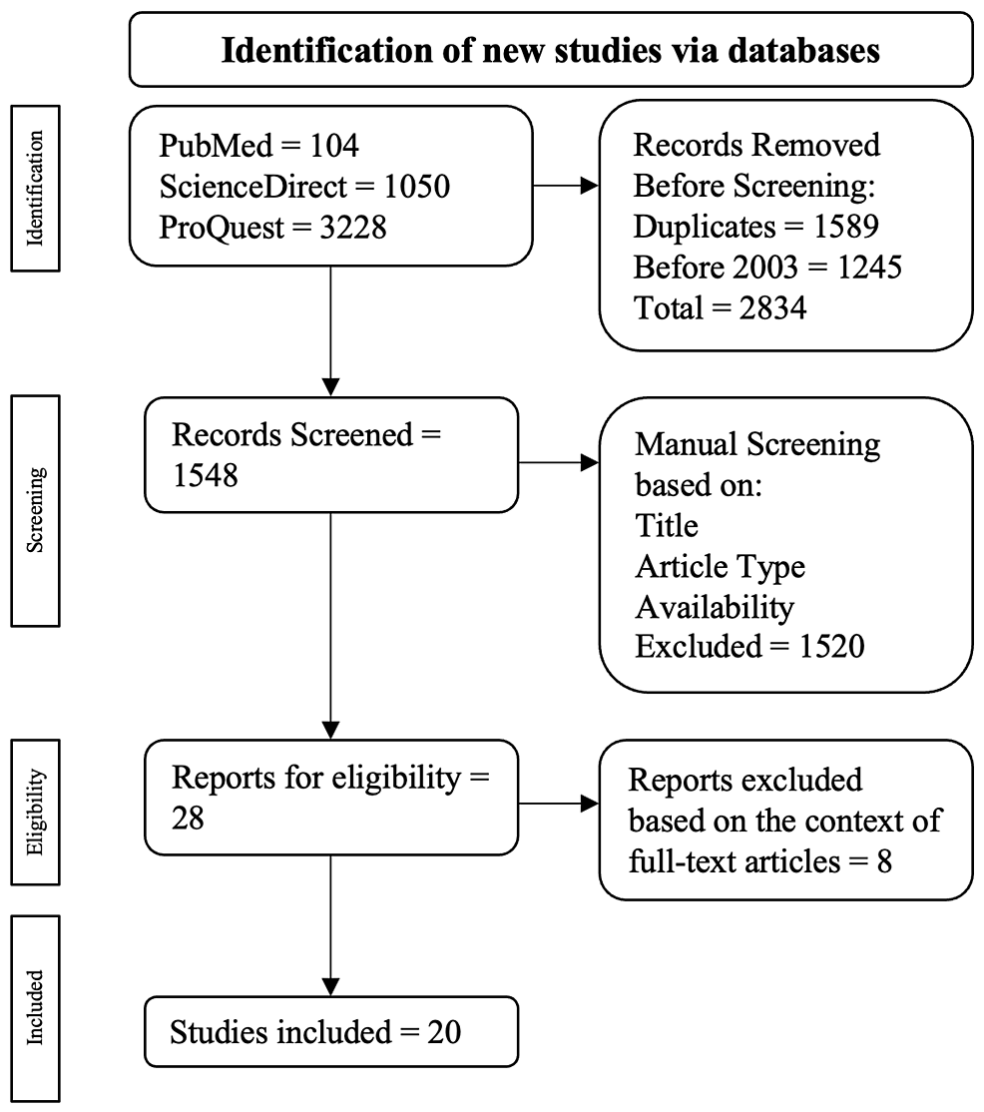
Algorithm employed based on the inclusion and exclusion criteria The flowchart was adapted to PRISMA guidelines [7].

Exclusion criteria were based on articles written not in any language other than English, no full-text availability, and duplications. The steps toward the procurement via the presented inclusion and exclusion criteria are depicted in Figure 1.

Bias

This article's overall rating of bias is moderate according to the GRADE (grading of recommendation, development, and evaluation) scale due to the inclusion of case reports centered around a single patient. All publications used in this review underwent a GRADE scale evaluation.

Results

A total of 4382 publications were populated: 104 were from PubMed, 1050 were from ScienceDirect, and 3228 were from ProQuest. Among the exclusions, 1589 were duplicate publications and 1245 were published before 2003. This led to 2834 publications being excluded in the automatic screening process, resulting in 1548 publications left for manual screening. Publications were then surveyed manually based on their title, study type, and full-text availability, leading to 28 publications being elected for full-text examination. Ultimately, 20 publications were selected.

According to a survey, women’s preference for TVA was 43%, with one reason for preference being a decrease in invasiveness. There were concerns regarding possible complications, length of recovery time, pain, and postoperative reproductive and sexual function. There are no indications for this route besides incidental findings during hysterectomy, but exclusion of patients has been made. Patients with complicated appendicitis, multiple abdominal surgery, or gynecological surgery were excluded from undergoing TVA. TVA has a relatively standard approach with the patients in the lithotomy position, umbilical incision to create a pneumoperitoneum at 13-15 mmHg, incision into posterior fornix, and performing the appendectomy. Variations occurred in the preferred method of the actual appendectomy, the appendix removal, and the posterior fornix incision closure. TVA has been shown to reduce postoperative complications such as wound infection, leading to reduced postoperative pain, shorter hospital stays, and quicker return to work. The need for analgesics decreased significantly; however, some physicians still placed women on patient-controlled analgesia (PCA) pumps. When complications did occur, the rates were low. Still, they ranged in severity from urinary tract infection or retention and vaginal cuff granulation to intra-abdominal abscesses and intra-operative bleeding of the appendix vessel. All patients were properly managed and returned to their pre-surgery health status. Studies evaluated in this review are summarized in Table 1.

**Table 1:** Summary of articles analyzed in this review per PRISMA guidelines TVA, transvaginal laparoscopic appendectomy; NOTES, natural orifice transluminal endoscopic surgery; LA, laparoscopic appendectomy; TGA, transgastric appendectomy

	Author	Country	Design & Study Population	Findings	Conclusion
1	Shin et al., 2010 [26]	Korea	Case report (n=1)	After TVA, the patient ate on day one and was discharged on day three without fever, wound infection, or urinary difficulties.	NOTES, specifically transvaginal, maybe a beneficial surgery to reduce recovery time with limited complications.
2	Solomon et al., 2011 [18]	USA	Retrospective cohort study (n=40)	The female sexual function index questionnaire showed no significant difference between LA and TVA, with no difference between pre- and postoperation	Despite the use of TVA, there is no difference in female sexual function index.
3	Knuth et al., 2014 [13]	Germany	Prospective cohort study (n=13)	There were no intra-operative complications and only two postoperative complications, including an infected hematoma and an abscess. One patient reported a vaginal fungal infection during their follow-up.	Overall, the transvaginal route is a feasible procedure with low intra-operative complications and is considered to be safe.
4	Mofid et al., 2013 [20]	Germany	Prospective cohort study (n=222)	One intra-operative complication of a bladder puncture and two cases were converted into LA. Two postoperative complications were a biliary fistula and an abscess in the pouch of Douglas. No pelvic pain, dyspareunia, or sexual dysfunction was reported.	The transvaginal route is still considered to be an appropriate method in NOTES.
5	Nezhat et al., 2009 [21]	USA	Retrospective cohort study (n=42)	All procedures were successful; no intra- or postoperative complications were reported.	The transvaginal method for appendectomy is deemed to be effective and results in acceptable outcomes.
6	Noguera et al., 2010 [15]	Spain	Case series (n=10)	All patients had successful operations with no complications during or after the surgery.	TVA shows that it is an appropriate procedure for intra-abdominal infections.
7	Palanivelu et al., 2008 [22]	India	Prospective cohort study (n=6)	Only one patient had a successful operation, with the remaining five being converted to LA or needing laparoscope assistance.	TVA does have satisfactory outcomes, but further studies are needed to confirm.
8	Panait et al., 2013 [23]	USA	Retrospective cohort study (n=107)	No major complications were reported, and no conversions were made.	Compared to laparoscopy, the transvaginal approach is safe, with good postoperative outcomes and better cosmetic outcomes.
9	Perez et al., 2011 [16]	Cuba	Retrospective cohort study (n=8)	No postoperative complications were reported. Only two patients required analgesics postoperatively. Five patients were discharged before 24 hours, and the remaining three at 48 hours.	Compared to laparoscopy, TVA is safe and has better aesthetic results. Further studies are needed to confirm the advantages.
10	Roberts et al., 2012 [17]	USA	Prospective cohort study (n=S42)	Statistically significant differences were seen in mean postoperative morphine use, return to normal activity, and return to work in comparison between TVA and LA. Four complications were reported, such as intra-abdominal abscess and urinary retention.	Compared to LA, TVA is safer, less painful, and quicker recovery times.
11	Tian et al., 2014 [19]	China	Case series (n=10)	No major intra- or postoperative complications occurred. The actual appendectomy took 21-34 min, and patients were discharged within four days following surgery.	TVA is a safe and feasible modification of the LA.
12	Wood et al., 2014 [24]	USA	Case series (n=102)	One complication occurred that resulted in an intra-abdominal abscess. Minor complications consisted of urinary retention.	As TVA begins to develop, complications will occur but can be learned about, leading to improvement of TVA. There also needs to be a specialization of laparoscopic instruments for transvaginal access.
13	Zorron et al., 2010 [27]	USA	Case series (n=362)	TVA was done in 10.2% of patients, and the only complication noted was a minor hemorrhage of the appendix vessel. TVA was significantly shorter than TGA and had fewer complications.	TVA may prove to be safer and more feasible than TGA.
14	Arezzo et al., 2013 [9]	Europe	Case series (n=5)	TVA was done in a significantly shorter time than TGA, with no complications observed in the TVA patients compared to the TGA group	TVA may be a safe preferred choice when the length of the procedure is considered, but no statistically significant evidence was found showing the benefit of TVA over TGA and small sample size.
15	Bernhardt et al., 2015 [10]	Germany	Prospective cohort study (n=10)	TVA was compared to LA. Overall, procedure time was less in laparoscopy. The only postoperative complication recorded was an abscess formed in a laparoscopic patient. Statistically significant advantages in the TVA were recorded with the use of postoperative questionnaires looking at activity in postoperative days one to 14, postoperative pain day one, general health conditions, and quality of life day three postoperation.	Flexible TVA appears to be a safe procedure that reduces postoperative recovery time and overall quality of life.
16	Chen et al., 2014 [11]	China	Prospective cohort study (n=5)	Five chronic appendicitis patients were selected to undergo a gasless TVA with concurrent hysterectomy. The appendectomy portion of the procedure was uncomplicated in all patients and took 20-30 min with minimal blood loss. Discharge was three days postoperation with no scar.	TVA with gasless laparoscopic after vaginal hysterectomy is a safe and feasible modification of an established procedure with acceptable outcomes.
17	Jategaonkar et al., 2020 [14]	India	Retrospective cohort study (n=18)	A modification for vaginal access to improve hybrid NOTES procedures was implemented. No procedure is needed for additional abdominal trocar or analgesia beyond six hours. The average procedure time was 27.5 min, and the hospital stay was 18.5 hours. Only one patient had postoperative spotting.	The investigated method is recommended because it is safe and has the potential to improve overall results, but a larger volume randomized trial is needed to confirm.
18	Khan et al., 2016 [12]	China	Cohort study (n=16)	16 TVAs using the hybrid NOTES technique with a single umbilical trocar were done. All procedures were successful with no intra- or postoperative complications or complaints for two years follow-up.	Compared to LA, hybrid NOTES can be done safely with less postoperative pain, lower cost, and shorter hospitalization.
19	Bingener et al., 2011 [8]	USA	Survey (n=409)	The transvaginal approach was acceptable for tubal ligation to 59%, acceptable for appendectomy in 43%, and for cholecystectomy to 41% of women. Preference on vaginal approach was due to decreased invasiveness, recovery time, and pain. Concerns were complications, pain, infection, and recovery time.	Keeping the concerns of the women in mind when considering the vaginal approach for various surgeries, approximately half are open to utilizing this approach while others have high concerns for scarring, pain, and recovery time leading them to prefer the standard laparoscopic approach.
20	Yagci et al., 2014 [25]	Turkey	Case report (n=1)	Operating times were within 75 min, and the patient was discharged 16 hours following the surgery with an uneventful stay. The patient also did not require analgesic medications.	Morbid obesity may not constitute as an exclusion or obstacle for the use of TVA.

Discussion

Patient’s Perceptions of Transvaginal Laparoscopic Appendectomy

While natural orifice surgeries have become increasingly popular and some are implemented regularly, they may still seem strange to the general population. One study evaluated women’s perception of transvaginal surgeries, including TVA. 43% of women found that TVA was an acceptable approach to LA, with one factor toward preference being a decrease in invasiveness at 14.4% [8]. Some women prefer this approach due to the proposed recovery time, minimal scarring, reduced pain, and entry location relative to what is being removed [8]. However, there were concerns proposed, such as possible complications, possible increased levels of pain, length of recovery time, and postoperative sexual and reproductive function [8]. Those agreeable with TAV tended to be younger, have a high educational status, and have fewer children [8].

Indications

Indications for the surgery had a common theme throughout publications. Some surgeons employed the surgery to investigate the safety and benefits of the NOTES procedure and determine if it was more minimally invasive than conventional LA [9,10]. Surgeons also preferred to implement this procedure to avoid abdominal wall incisions, eliminate scaring, reduce postoperative wound infections, reduce the occurrence of postoperative abdominal hernia seen in conventional LA, and reduce postoperative pain [10-12]. Generally, trocar hernias are possible complications of abdominal wall trauma but can be avoided by entering the abdomen via NOTES [13]. Another indication is that submucosal tunnels limit the need for expensive gadgets to complete the procedure [14].

Women who were included in the study to receive TVA had acute appendicitis, chronic appendicitis when there was no filling of the appendix during barium enema, more than one attack of acute appendicitis, presence of a fecalith, delivery of at least one child, between 18 and 65 years old, and non-perforated appendicitis [15-19]. The average age of women who underwent TVA was 37.4, with a mean BMI of 30.63 mg/k^2^ [16,20-24]. Women also tended to be young, non-pregnant, married with families, thin-built, and did not have pelvic inflammatory disease [22]. Women were excluded if they had retrocecal appendix, were pregnant, had past abdominal or gynecological surgery, had complicated appendicitis like appendicular abscess, were virgins, had gynecological infections, aged <18 or >65, had pelvic adhesions, had prior malignancy, chemotherapy, or BMI >35 kg/m^2^ [13,16-20,22]. Despite the BMI limitations, two publications performed surgery on women with a BMI over 35 kg/m^2^ to test whether it is a true contraindication [23,25]. Another two publications only performed TVA in patients undergoing laparoscopic vaginal hysterectomy as they were already performing a vaginal surgery [19,21].

Transvaginal Laparoscopic Appendectomy Procedure

The application of the TVA was relatively consistent across studies, with minor variation in the surgeon's preference for objects like sutures or techniques in suturing. Before surgery, patients receive prophylactic antibiotics such as cefuroxime, metronidazole, amoxicillin, clavulanic acid, gentamicin, or cefazolin [11,14-16,19-21,26]. Patients were then induced with endotracheal anesthesia and placed in a low lithotomy position in Allen stirrups [11-13,16,18,19,21,22,25-27]. One study, however, chose to put patients in the 30° Lloyd-Davies position with hands tucked along their sides [14]. The vagina was then cleaned with appropriate anti-septic fluid before surgery [20]. Initial steps involve making a 5 mm incision at the umbilicus to inject a 5 mm trocar. Within the trocar, a Veress needle was used to create a pneumoperitoneum between 13 and 15 mmHg, depending on the study [10,12-16,19,21-23,25-27]. In two studies, the surgeon decided not to use any umbilical or abdominal ports [11,19].

Once abdominal ports were placed, a speculum was used to view the transvaginal entry site [13]. The cervix was then retracted anteriorly with a single-tooth tenaculum or a deep cavity retractor, and depending on the difficulty, a uterine probe was then used to bring the uterus into anteflexion [13,14,17]. Once the cervix was open 12-15 mm, incision by a Bovie electrocautery was used to open the posterior vaginal fornix, and a trocar with a camera was inserted to allow for identification of the appendix, masses, or extensive adhesion [9,12-14,16-18,20,22,23,25-27]. Patients who had a larger uterus required a laparoscopic colpotomy incision, and if the patient had an adnexal infection and appendicitis, only the 5 mm umbilical trocar was used [15,21]. The appendix was then held using either a grasper, rat-tooth biopsy forceps, or curved grasping forceps to hold the appendix [13,16,22,25,26]. If the appendix was retrocecal, the retroperitoneal structures, bowel attachments, and mesoappendix were freed [21].

The mesoappendix was prepared with coagulation forceps, a dent was then created in the mesoappendix, and it was electrodesiccated or divided by either Ligasure, a Maryland dissector, or an ultrasonic scalpel in coagulation mode [10-12,16-19,21,22,25-27]. The sealing and clipping of the appendix at the base varied between studies, with some studies using Endoloop and Endosnare to ligate the base, ultrasonic scalpel, or Hem-o-lock clips [9-12,16,19,22,26]. One study placed a stapler across the base of the appendix to remove it in one application, and the appendix was taken out using the colpotomy incision [21]. The removal of the appendix was through the vaginal incision, but some studies used a specimen retrieval bag while others did not [13,15-22,27]. Once there was hemostasis confirmed and the integrity of the bowel was inspected, the vaginal incision was closed with resorbable sutures such as chromic catgut stitches, Vicryl, or Polysorb sutures [12-23,26].

At completion, some patients received iodine-soaked packing to prevent bleeding and fistula formation [12,14]. In a series of cases, some patients received T-shaped tubes as vault drainage and were placed through the vagina and removed 40 hours later [19]. For postmenopausal patients, they received estrogen suppositories for five days [20]. In one surgery, antibiotic suppositories were given to be inserted for approximately a week [26]. All patients were advised to avoid sexual intercourse for two to four weeks [15,20,22]. Following discharge, patients were advised or immediately scheduled for multiple follow-ups up to a year following surgery [15-17,22].

Positive Outcomes Following Transvaginal Laparoscopic Appendectomy

The operation time of TVA ranged from 25 to 103.5 min, including in patients with BMI >35, with the actual appendectomy taking only 5 to 10 min in one study [9,11-17,20-23,25-27]. Following TVA, some patients received no analgesics as the pain was reported as 0, others received either one dose of parenteral analgesics, and others received PCA morphine for 12 hours postoperation [10,12,14,17,22,25]. When pain was reported, standard steps toward analgesia were employed per the surgeon’s preference, and those on PCA had a mean usage of 8.7 mg of morphine [16,17]. Most studies reported no intra-operative complications, such as excess blood loss or transvaginal gas leakage, nor the need to convert to a conventional LA [11,12,14,19,25]. Arezzo et al. reported that 67% of their TVAs needed additional transabdominal laparoscopes for better tissue manipulation [9]. Even with patients whose BMI was >35 mg/k^2^, there was no need to convert to a standard LA or open appendectomy [23].

After patients were moved from recovery to a standard room, no fever, urinary difficulties, or pelvic pain were reported [19,25,26]. Surgeons reported no wound infection, no postoperative leakage related to colpotomy, no hemorrhage related to colpotomy, and no vaginal cuff infection [12,19,25]. The majority of patients, including those with a BMI >35 kg/m^2^, had no postoperative complication or mortality [9-11,13,19,22,23]. Roberts et al. observed that no patients complained of postoperative dyspareunia and no statistically significant difference in sexual function [17]. Within 12 hours, patients were able to consume water, and in some studies, patients ate within 24 hours, but the typical diet was not achieved until 48 hours later [19,21,22,25,26]. The physical condition on day one postoperative was significantly better than LA, with all patients in one study walking within four hours following surgery [10,14]. The length of hospital stay ranged from 0.2 to 3 days at the most across studies similar or reduced in some cases compared to LA with no changes in patients with a BMI >35 kg/m^2^ [9,11,12,14-17,19-23,25,26]. Patients requiring antibiotic treatment had extended hospital stays to complete the therapy [15].

No patients needed narcotics at or after discharge [11,14,19,25]. TVA patients had a significant statistical advantage of resuming postoperation activities based on questionnaires investigating the desire to be discharged, first shower, first bike ride, first defecation, resuming household activity, sports hobby and cultural activities, and overall well-being, which showed a return to activities as early as one day, and all patients by 10 days postoperation [10,12,14,17]. Within two weeks following surgery, all surgical wounds were well healed with no reports of general or surgical wound complications, scarring, hernias, or complaints of dyspareunia [12,15,20,22,25,26]. TVA also led to no significant difference in the overall women's sexual function index questions, with women returning to normal sexual activity two weeks later [18-20,22]. With such success, Mofid et al. created a survey asking for recommendations for TVA versus LA, and 99% of patients recommended TVA [20].

Complications Following Transvaginal Laparoscopic Appendectomy

Across the board, TVA had a low complication rate, with one study reporting an 8.1% complication rate [21,27]. Some cases required another 2-12 mm trocar, including a drainer, linear stapler, and larger clips [20]. In some instances, the TVA had to be converted to an LA, with Wood et al. calculating it happened to 8% of TVAs [14,17,24]. This occurred from dense pelvic adhesions and the inability to maintain the pneumoperitoneum [14,17,24]. A few patients reported fevers, with one patient reporting a fever of up to 101.8°F postoperation [21]. In one instance, an intra-operative complication of a bladder puncture occurring during the entry of the trocar through the posterior vaginal wall occurred [20]. Other complications that did occur were intra-abdominal abscesses, intra-operative bleeding of the appendix vessel, the need for intra-operative drain placement, infected pelvic hematoma, postoperative abscess in the cecum, umbilical incision cellulitis, urinary retention, urinary tract infection, and vaginal cuff granulation [11,13,17,19,21,22,24,27]. Solomon et al. did observe that compared to other studies, TVA had significantly decreased sexual function postoperatively [18].

A limitation to this study is the relatively small sample size of the studies, due to TVA still in the phase of being evaluated. NOTES is not a new concept in the last two decades, as umbilical approaches are frequently used. However, other orifices have little studies on them because they are relatively abnormal, which may explain the reduced sample size. Another limitation of this study is that it possibly used case reports, as they consist of only one patient. Still, these studies were correlated with experimental studies and case series that report a statistical trend over the popular. These case reports, however, highlight possible abnormalities that have not been reported yet. Because the sample size of the experimental studies is relatively small, it does elevate the extent of the reported symptoms. Ideally, future research should achieve larger sample sizes and compare them to conventional LA. Also of interest is the possible long-term side effects that may disrupt fertility.

## Conclusions

TVA has a relatively standard approach with prophylactic antibiotics, positioning the patient in the lithotomy position, an umbilical incision made for a pneumoperitoneum, incision and trocar placement at the posterior fornix, standard appendectomy, and suturing the posterior fornix following confirmation of hemostasis. The patient and physician observed minimal complications, with a quick resumption of diet. The hospital stay was reduced, with patients requiring antibiotics requiring a longer stay. There was also a significant reduction in pain management; in some patients, no pain was reported, leading to reduced/no analgesics. Sexual function and activity were resumed by two weeks postoperation. When complications did occur, they included conversion to LA, intra-abdominal abscess, intra-operative bleeding of the appendix vessel, urinary retention, urinary tract infection, and vaginal cuff granulation.

There is always a need to improve and perfect a technique for the betterment of the patient undergoing the procedure. TVA offers this possibility, with some studies reporting better results than the conventional LA. However, due to its uniqueness, few studies have been done evaluating the TVA and they consisted of a small population. Future studies should try to implement a larger population size so that a full evaluation can be done and reproducibility can be seen to increase significance. TVA may also pose a long-term risk regarding reproductive functions and fertility capabilities; however, this has not yet been evaluated, and current research should include patient follow-up in women of reproductive age.
